# Oil Contact Angles in a Water-Decane-Silicon Dioxide System: Effects of Surface Charge

**DOI:** 10.1186/s11671-018-2521-6

**Published:** 2018-04-19

**Authors:** Shijing Xu, Jingyao Wang, Jiazhong Wu, Qingjie Liu, Chengzhen Sun, Bofeng Bai

**Affiliations:** 1 State Key Laboratory of Enhanced Oil Recovery, Research Institute of Petroleum Exploration and Development of PetroChina, Beijing, 100083 China; 2 State Key Laboratory of Multiphase Flow in Power Engineering, Xi’an Jiaotong University, Xi’an, 710049 China

**Keywords:** Oil wettability, Silicon dioxide surface, Surface charge, Molecular dynamics

## Abstract

Oil wettability in the water-oil-rock systems is very sensitive to the evolution of surface charges on the rock surfaces induced by the adsorption of ions and other chemical agents in water flooding. Through a set of large-scale molecular dynamics simulations, we reveal the effects of surface charge on the oil contact angles in an ideal water-decane-silicon dioxide system. The results show that the contact angles of oil nano-droplets have a great dependence on the surface charges. As the surface charge density exceeds a critical value of 0.992 e/nm^2^, the contact angle reaches up to 78.8° and the water-wet state is very apparent. The variation of contact angles can be confirmed from the number density distributions of oil molecules. With increasing the surface charge density, the adsorption of oil molecules weakens and the contact areas between nano-droplets and silicon dioxide surface are reduced. In addition, the number density distributions, RDF distributions, and molecular orientations indicate that the oil molecules are adsorbed on the silicon dioxide surface layer-by-layer with an orientation parallel to the surface. However, the layered structure of oil molecules near the silicon dioxide surface becomes more and more obscure at higher surface charge densities.

## Background

Enhanced oil recovery (EOR) is becoming more and more important with the growing difficulties in the extraction of crude oil from oil reservoirs [1, 2]. Among various EOR techniques, water flooding is a dominating approach owing to its low cost and high efficiency. Water flooding is a multiscale process, including either the viscous fingering processes in the porous reservoirs or the two-phase flow processes that the crude oil is displaced by the injected water in microfluidic channels. As a result, water flooding is controlled by various factors from pore scale to reservoir scale. Owing to the large surface-to-volume ratios of the oil reservoirs and the low capillary numbers of oil displacement process, the wettability of oil and water on the porous rock surfaces has a great impact on the oil-water two-phase flows in oil reservoirs. Therefore, wettability of rock surfaces is very crucial for the oil recovery efficiency [3–5].

Generally, the water-wet rock surface is beneficial for the oil displacement; however, the rock surface initially appears as oil-wet for the adsorption of polar molecules from crucial oil and others. Thus, the wettability alteration of rock surfaces from oil-wet to water-wet is very important for the EOR techniques. In the water flooding, people usually artificially add some chemical agents (e.g., surfactants, polymers, ions, nanoparticles) into the injected water to realize the wettability alteration [6–11]. For example, the dissolved surfactants in injected water can alter the wettability of reservoir rocks to a more water-wet state; it is generally accepted that the ion-pair formation and the adsorption of surfactant molecules through the interactions with the adsorbed crude oil components on rock surfaces are the two main mechanisms responsible for the surfactant-induced wettability alteration [12]. For the low-salinity water flooding, the double-layer expansion upon reducing the salinity and the multicomponent ion exchange are two specific competing mechanisms for the wettability alteration of sandstone reservoirs [13, 14]. While for the nanoparticle-based water flooding, the self-layering and low-dimensional structuring of nanoparticles in the three-phase contact line region exert a structural disjoining pressure to the oil phase and finally promote the oil-wet rock surfaces altering to a water-wet state [15–18].

Regardless of the variety of water flooding techniques, the essential mechanism of wettability alteration is the change of the three-phase interactions among water-oil-rock induced by the injected chemical agents. For the water-oil-rock interactions, the electrostatic interactions play a profound role in the wettability alteration, because the chemical agents can adsorb onto the rock surfaces and subsequently change the surface charges. For instances, the divalent cations can promote the binding of acidic oil components to negatively charged rock surfaces and accordingly modify the electric potential of rock surfaces from negative to positive [19–22]. Meanwhile, the chemistry of water itself could affect the charges on rock surfaces [23]. Thus, the surface charge is definitely one of the most important factors affecting the wettability of rock surfaces. Puah et al. [24] showed that the surface charge affected both the static wettability and wetting kinetics of solid surfaces. They further confirmed that the static contact angle decreased above and below the point of zero surface charge in a Lippman-like manner, while the dynamic contact angle data can be well described by the molecular kinetic theory.

Because the three-phase wettability is directly related to the molecular interactions among water-oil-rock, a molecular insight study is very necessary to reveal the effects of surface charge on the wettability of rock surfaces [25, 26]. In this paper, we build a silicon dioxide surface to model the silicate rock surfaces and study its three-phase wettability with considering the surface charges by using the molecular dynamics (MD) simulation method. The effects of surface charge and their underlying mechanisms are expected to be revealed from the aspects of molecular number density distributions, molecular structure on rock surfaces, etc.

## Methods

### Simulation System

**Fig. 1:**
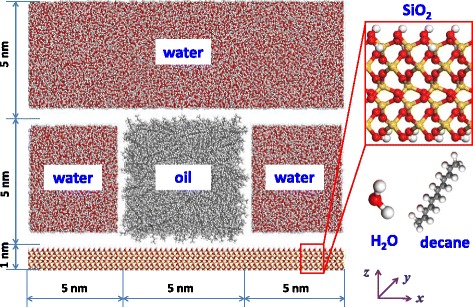
Schematic illustration of the simulation system. Side view of the simulation system with an oil cube surrounded by water on the top of silicon dioxide substrate (left) and atomic views of decane and water molecules (right)

We study the three-phase wettability in a model system, where the oil is modeled as decane, while the rock is modeled as silicon dioxide. In particular, the silicon dioxide surface can well represent the sandstone reservoirs [11, 27, 28]. To calculate the oil contact angle, the oil is enclosed in water as a cylindrical droplet (see Fig. 1). For the cylindrical oil droplet, the effects of line tension on the calculation of contact angle can be eliminated. To build the silicon dioxide substrate, a set of trigonal unit cells of α-quartz is assembled in the [100], [010], and [001] directions, respectively. Then, the α-quartz crystal is transformed from a rhombohedron to a cube with a size of 15 × 15 × 1 nm^3^. Much attention must be paid to obtain a realistic surface structure; the same as the procedure of Puibasset et al. [29], the crystal is firstly cut along the (111) crystallographic face and the silicon atoms in the incomplete tetrahedrons are removed. Then, the nonbridging oxygen atoms, which are bonded to only one silicon atom, are saturated with hydrogen atoms. Namely, the top and bottom sides of the silicon dioxide substrate are both saturated with hydrogen atoms. Therefore, we simply alter the surface charge by adding partial charges into the top-hydrogen atoms on the silicon dioxide surface, while the entire system is kept neutralized by adding equivalent partial charges with inverse values into the bottom-hydrogen atoms. The structures of decane and water molecules are also shown in Fig. 1.

As shown in Fig. 1, the water and oil are initially arranged on the top of the silicon dioxide substrate and the oil cube is surrounded by three water cubes with different sizes. The oil cube has a dimension of 5 × 15 × 5 nm^3^ and is located at the central part of the substrate. The dimensions of the left- and right-side water cubes are the same as that of the oil cube, while that of the top-side water cube is 15 × 15 × 5 nm^3^. The number of water and decane molecules is determined by the sizes of the cubes and the water and oil densities under the condition of 300 K and 1 bar. The densities of water and oil are 1.000 and 0.725 g/cm^3^, respectively; accordingly, the number of water and decane molecules is 58,319 and 1150, respectively. The arrangement of molecules is implemented in the Material Studio software; originally, they are randomly distributed in the special boxes. Then, a calculation of energy minimization is conducted to optimize the molecular geometry. As the simulation reaches to an equilibrium state, the oil box evolves into a cylindrical droplet, while the three water boxes can merge together forming a continuous phase surrounding the oil droplet.

### MD Model

**Fig. 2:**
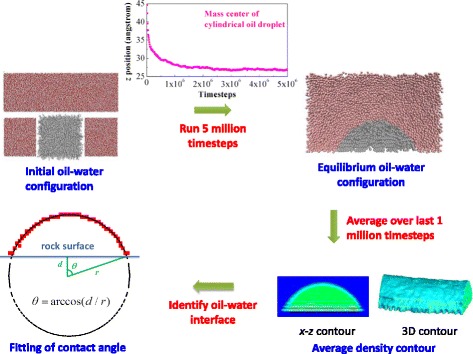
Calculation procedures for the contact angles of cylindrical oil droplets

Our MD simulations are performed using the platform of LAMMPS (large-scale atomic/molecular massively parallel simulator). The simulations are conducted in a NVT ensemble with a temperature of 300 K. The simulations run totally for 5 million timesteps with a time step of 1 fs. The atomic coordinates are recorded every 10,000 timesteps to observe the evolution of oil droplets and finally calculate the contact angles. The system can reach to an equilibrium state in around two million timesteps, as shown in Fig. 2. Thus, the atomic coordinates during the final one million timesteps are adopted to average the molecular number density distributions. Periodic boundary conditions are applied in the *x*- and *y*-directions, while the reflective boundary condition is applied in the *z*-direction. During the simulations, the atoms of bulk SiO_2_ are fixed, but the surface O and H atoms are flexible under the control of Nose-Hoover thermostat.

The atomic interactions among water-decane-silicon dioxide are all modeled by 12-6 Lennard-Jones potential coupled with a polar term to consider both the Van der Waal and Coulombic forces [30–32], as follows:

1 $$ \phi \left({r}_{ij}\right)=\left\{\begin{array}{ll}4\varepsilon \left[{\left(\frac{\sigma }{r_{ij}}\right)}^{12}-{\left(\frac{\sigma }{r_{ij}}\right)}^6\right]+\frac{Cq_i{q}_j}{\chi {r}_{ij}}& \left({r}_{ij}<{r}_{\mathrm{cut}}\right)\\ {}0& \left({r}_{ij}\ge {r}_{\mathrm{cut}}\right)\end{array}\right. $$

where *ε* is the energy parameter, *σ* is the length parameter, *q*_i_ and *q*_j_ are the charges on atoms *i* and *j*, *C* is the electrostatic constant, and *χ* is the dielectric constant. *R*_cut_ is the cutoff distance for the Van der Waal and short-range electrostatic forces. The cutoff distance is set to be 10 Å. In the simulations, the long-range electrostatic forces are considered by using the particle-particle particle-mesh method. To obtain the potential parameters in Eq. (1), the TIP3P model is adopted for H_2_O molecules [33]; for decane molecules, the OPLS-AA force field is adopted [34]; for the silicon dioxide substrate, the CVFF force field is adopted [35]. The non-bonded potential parameters used in the simulation system are listed in Table 1. The Lorentz-Berthelot rule is used to obtain the potential parameters between crossing atoms. The bond interactions and dihedral interactions between quadruplets of atoms in a single molecule are also considered properly. For the bond interactions, the harmonic model is applied, while for the dihedral interactions, the OPLS potential model is applied [36].

**Table 1:** Non-bonded potential parameters used in the simulation system

	Atom	Charge (e)	*σ* (Å)	*ε* (10^−3^ eV)
Water	O	− 0.834	3.150	6.611
	H	0.417	0.400	1.999
Decane	C_1*_	− 0.120	3.500	2.864
	C_2*_	− 0.180	3.500	2.864
	H	0.060	2.500	1.302
Silicon dioxide	Si	1.100	4.150	4.030
	O_bulk_	− 0.550	3.470	2.340
	O_surface_	− 0.675	3.470	5.290
	H	0.400	1.085	0.650

*1 represents the carbon atoms bonded with two hydrogen atoms; 2 represents the carbon atoms bonded with three hydrogen atoms

### Calculation of Contact Angle

The wettability of the silicon dioxide surface is characterized by the oil contact angle, which is obtained based on the density distributions of oil molecules in the nano-droplets. At the earlier stage of the simulations, the oil droplet evolves from a cube to a semi-cylinder and reaches to an equilibrium state; this equilibrating process usually takes two million timesteps, which can be confirmed from the time variation of the mass center of oil droplets. At the equilibrium state, the coordinate of the mass center keeps constant in the *z*-direction. Because the density distribution of oil molecules need to be averaged for the equilibrium oil-water configurations, to be doubly sure, we only collect the atomic coordinates in the final one million timesteps to obtain the density distributions. To average the molecular number densities, the oil/water zone is divided into many regular cubic cells with a size of 2 × 2 × 2 Å^3^. By timely averaging the number of atoms appeared in each cell, the 3D contour and the 2D contour in the *x*-*z* plane of the density distributions of oil nano-droplets can be obtained. It is noted that the 2D contour in the *x*-*z* plane is obtained by further averaging the 3D contour in the *y*-direction. With the 2D contour, the oil-water interface is identified based on the following rule; at the interface, the number density of oil atoms in the interface-cells is half of the density in the bulk-phase cells of oil droplets (*ρ*_b_). Considering the interface thickness, the locations of such cells are defined as interface if the densities in the cells are in the range from 0.2*ρ*_b_ to 0.8*ρ*_b_. By using the above method, the discrete points on the interface can be identified. Finally, we obtain the interface profile by fitting these discrete points as a circle and calculate the contact angle of the oil droplets. In the fitting, the center position (*x*_0_, *z*_0_) and the radius *r* of the circle are necessarily obtained. The contact angle *θ* can be calculated as *θ* = arccos(*d*/*r*), where *d* is the distance between the circle center and the base line of rock surface in the *z*-direction (*d* = *z*_b_ − *z*_0_, *z*_b_ is the *z*-position of the base line, *z*_b_ = 12.16 Å). If the distance is a negative value, the circle center is on the top of rock surface and the contact angle definitely exceeds 90°. The entire calculation procedures of contact angle are explicitly depicted in Fig. 2. To obtain the contact angle in such a way, it is enough to analyze the cylindrical oil droplet in the Cartesian coordinate system.

Owing to the lack of effective data on the contact angles of water-surrounded oil droplets on silicon dioxide substrates, we validate our simulation model by comparing the contact angles of spherical water droplets on silicon dioxide substrates. We conduct an individual simulation to obtain the contact angle of a water nano-droplet with 4179 molecules. It is noted that in the validation simulation, the hydroxyl groups on the surface of silicon dioxide substrates are fixed as other representative works done. The measured contact angle is 7.8°, which is in good agreement with that in the work by Pafong et al. [37]. They reported a water contact angle of 7.0°, which presents a relative deviation of only 10.3%. This good agreement indicates that our simulation model and method are reliable.

## Results and Discussion

### Contact Angle

**Fig. 3:**
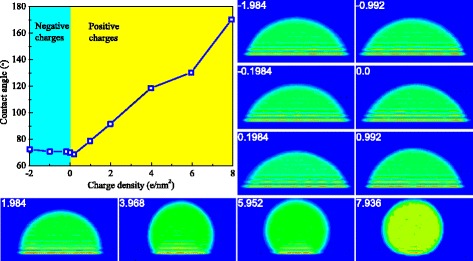
Variation of contact angles with the surface charge density. The contour images of oil droplets at different surface charge densities are also inserted

By calculating the contact angles of cylindrical oil droplets, the variations of contact angle with surface charge density can be obtained, as shown in Fig. 3. It can be clearly seen from this figure that the contact angle has a great dependence on the surface charge density; from the negatively charged surface to positively charged surface, the contact angle of oil droplets increases significantly. This means that the rock surface alters from oil-wet state to water-wet state as the surface charge increases from negative values to positive values. The average density contours in the *x*-*z* plane at various surface charge densities are also inserted in this figure to have an intuitive understanding of the variation of surface wettability.

We can also find from Fig. 3 that the rock surface presents a very obvious water-wet state as the surface charge density increases up to 0.992 e/nm^2^. Below this critical value, the variation of contact angle with surface charge density is not obvious; beyond this critical value, the contact angle reaches up to 78.8° and the water-wet state is very apparent. It is believed that the variation of contact angle with surface charge is caused by the change of the intensions of water-silicon dioxide and oil-silicon dioxide, because the surface charge affects the interactions between water-silicon dioxide and the interactions between oil-silicon dioxide. It should be particularly noted that the simulation results based on this ideal system cannot be directly cross-compared with the experimental studies for revealing the salinity-dependent wettability in the oil-water-rock systems. In the experimental studies, the cations in water phase may have the effect of modifying the electric potential of rock surfaces from negative to positive, but it is definitely not their only effect. The other potential effects of cations may include multi-ion exchange and double-layer expansion etc. [21, 38–40]. Therefore, it is totally different for the cases of changing the surface charge and changing the concentration of the cations in water. Accordingly, the variation trends of contact angles with the surface charge density and the concentration of cations may differ. In this simulation study, we only discuss the effect of partial charges on silicon dioxide surface on the three-phase wettability in a very ideal oil-water-rock system. These results cannot directly reveal the mechanisms of low-salinity water flooding and guide its applications in enhanced oil recovery, but they can provide basic understandings on how the surface charges affect the three-phase wettability. This study is still of significance because it reveals the profound roles of surface charges on the three-phase wettability in a water-decane-silicon dioxide system.

### Density Distribution

**Fig. 4:**
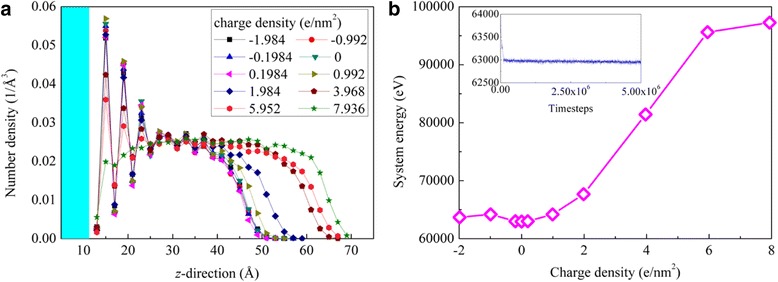
**a** Density distributions of oil molecules along the *z*-direction; **b** Time-averaging system energy at different surface charge densities

To reveal the mechanisms of the charge density-dependent contact angle, we analyze the density distributions of oil molecules along the height direction (*z*-direction). Figure 4a gives the average density of oil molecules in each layer with a width of 0.2 nm along the height direction. As seen from this figure, at the bottom of the cylindrical droplet (i.e., near the substrate surface), the oil density is high and presents fluctuation distributions; this phenomenon means that the oil molecules are adsorbed on the substrate surfaces in layered structure. At the top of the cylindrical droplet, the average density drops down to a zero-near value, because the arc-shaped top of the droplet makes the number of oil molecules in the parallel layers less and less as the layers approach to the top of the droplet. Meanwhile, we can find that, as the surface charges turn from negative values to positive values, the number density in the adsorption layers decreases gradually; this indicates that the adsorption intensity of oil molecules weakens with increasing the surface charges. The weakening adsorption of oil molecules further means a smaller contact area between substrate surface and oil droplet and accordingly a bigger contact angle of oil droplet. The increasing contact angles of the oil droplets can also be reflected from the variation of the heights of oil droplets with the surface charges. As seen from Fig. 4a, the height of oil droplet increases with increasing the surface charges; this means that at higher surface charges, the oil droplets are slimmer and the contact angles are bigger. The weakening adsorption of oil molecules at positive surface charges can be confirmed from the system energy of the water-decane-silicon dioxide systems. As shown in Fig. 4b, the time-averaging system energy increases with increasing the surface charges. The higher the positive system energy is, the weaker the attractions between oil molecules and silicon dioxide are. The inserted figure shows the time variations of the system energy for the neutral silicon dioxide surface.

**Fig. 5:**
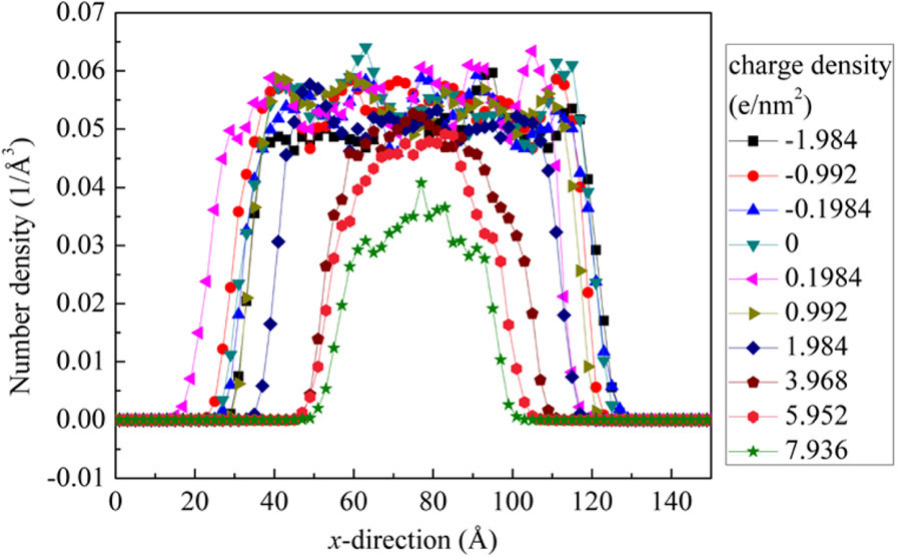
Density distributions of oil molecules along the *x*-direction in the *x*-*y* plane with a height of 1.9 nm in the *z*-direction

The variation of contact angle with surface charge can also be reflected from the molecular number density distributions along the *x*-direction. As shown in Fig. 5, the number density distributions along the *x*-direction in the *x*-*y* plane with a height of 1.9 nm in the *z*-direction presents a platform in the central part. The width of the platform is related to the cross-section area of the droplet in the *x*-*y* plane, while the height of the platform is related to the molecular number density in the droplets. The width and height of the platform vary distinctly at different surface charges, which correspond to the different shapes of cylindrical droplets and of course the different contact angles. For the lower contact angles, the platform is wider; for the higher contact angles, the platform is narrower. It is noted that the height of the platform for the surface charge density 7.936 e/nm^2^ is especially low, because near the silicon dioxide surface the density distributions have a great fluctuation (see Fig. 4a) and, in some cases, the molecular densities at the height of 1.9 nm are just located at the lows of the density distributions.

**Fig. 6:**
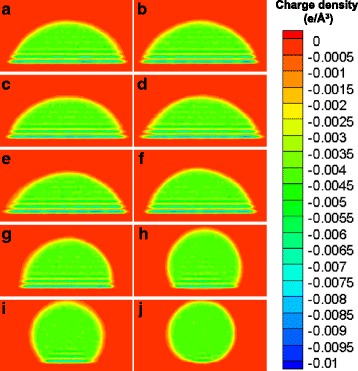
Negative charge density distributions inside the cylindrical oil droplets in the *x*-*z* plane. **a** − 1.984 e/nm^2^. **b** − 0.992 e/nm^2^. **c** − 0.1984 e/nm^2^. **d** 0.0 e/nm^2^. **e** 0.1984 e/nm^2^. **f** 0.992 e/nm^2^. **g** 1.984 e/nm^2^. **h** 3.968 e/nm^2^. **i** 5.952 e/nm^2^. **j** 7.936 e/nm^2^

We also obtain the density distributions of negative charges of the atoms inside the cylindrical droplets, as shown in Fig. 6. Basically, the negative charges present a very non-uniform distribution. In the zones adjacent to the silicon dioxide surface, the density of negative charges is extremely high, while in the zones far away from the silicon dioxide surface, the negative charges distribute uniformly. Meanwhile, in the high-density zones adjacent to the silicon dioxide surface, the negative charges distribute layer by layer. The layered distribution of negative charges is directly related to the layered structure of oil molecules near the silicon dioxide surface, because the negative charges directly associate with the carbon atoms in oil molecules. In addition, we can find that the density distributions of negative charges have a slight diversity at different surface charge densities. As the surface charge density increases, the layered distribution of negative charges becomes more and more obscure. This is related to the reduced contact areas of the nano-droplets with the silicon dioxide surfaces; at high surface charge densities, the contact areas are reduced and the layered structure of oil molecules becomes unapparent owing to the weakened solvation interactions.

### Molecular Structure

**Fig. 7:**
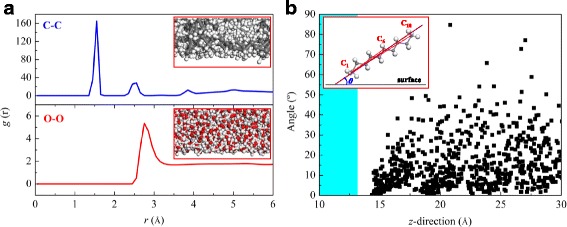
Molecular structures on the neutral silicon dioxide surface. **a** RDF distributions of oil and water molecules. **b** Orientations of oil molecules located at different heights in the *z*-direction

The molecular structures in the water-decane-silicon dioxide system are also very important for the understanding of the three-phase wettability. We try to analyze the molecular structures of oil and water from the radial distribution function (RDF, *g*(*r*) [41]) and the molecular orientations. Figure 7 gives the RDF distributions of oil and water molecules and the orientations of oil molecules on the neutral silicon dioxide surface. The RDF distributions of O–O and C–C atomic pairs are totally different (see Fig. 7a), namely, the RDF distributions of C–C atoms have more waves than those of O–O atoms and the peak values of the waves for C–C atoms are higher. Coupling with the physical meanings of RDF functions, it can be easily concluded that the oil molecules are strongly adsorbed on the silicon dioxide surfaces with several layers, while the water molecules are weakly adsorbed and only one adsorption layer appears. The adsorption phenomenon also can be seen from the inserted maps in Fig. 7a, where the equilibrium snapshots of oil and water molecules on the neutral silicon dioxide surface are displayed. The obvious adsorption layers of oil molecules can be confirmed from the number density distributions in Fig. 4a. Generally speaking, the layered structure of liquid molecules on a solid surface is related to the liquid-solid interactions. For the strong liquid-solid interactions, the liquid molecules near the solid surface distribute layer-by-layer along the direction normal to the surface, while for the weak liquid-solid interactions, the layered structure of liquid molecules is not very apparent [42]. In this study, the interactions between water and silicon dioxide are relatively weaker compared with those between oil and silicon dioxide. Thus, the layered structure of oil molecules near the solid surface is more obvious than that of water molecules.

In order to further reflect the orientations of the long-chain oil molecules on the silicon dioxide surfaces, we obtain the angles between oil molecules and silicon dioxide surfaces for the molecules located at different heights in the *z*-direction, as shown in Fig. 7b. The angle *θ* is defined as the crossing angle between the base line of the silicon dioxide surface and the central line of an oil molecule, where the central line is defined as the mean line of the connecting line between C1 and C6 and the connecting line between C1 and C10. The sequence number of carbon atoms in an oil molecule is started from one end side of the molecule, which is more adjacent to the silicon dioxide surface. The definition of angle *θ* is also inserted in Fig. 7b. As seen from this figure, the angles for the majority of oil molecules are very small, especially for the oil molecules near the silicon dioxide surface. This means that the oil molecules are adsorbed on the silicon dioxide surface with an orientation parallel to the surface. This phenomenon can also be confirmed from the inserted maps in Fig. 7a.

## Conclusions

Using MD simulations, we study the effects of surface charge density on the oil contact angles in a water-decane-silicon dioxide system. The results show that the contact angle of oil nano-droplets increases significantly as the surface charge increases from negative values to positive values. As the surface charge density exceeds a critical value of 0.992 e/nm^2^, the contact angle reaches up to 78.8° and the water-wet state is very apparent. The variation of contact angles can be confirmed from the density distributions of oil molecules along the *x*- and *z*-directions. The decreasing number density of oil molecules in the adsorption layers and the increasing height of oil droplet both mean a bigger contact angle of oil droplets at higher surface charge densities. Owing to the layered structure of oil molecules near the silicon dioxide surface, the negative charges adjacent to the silicon dioxide surface inside the oil droplets also distribute layer-by-layer. As the surface charge density increases, the layered distribution of negative charges (oil molecules) becomes more and more obscure due to the weakened oil-silicon dioxide interactions. The RDF distributions of oil and water molecules and the molecular orientation distributions also show that the oil molecules are adsorbed on the silicon dioxide surfaces layer-by-layer with an orientation parallel to the surfaces. These results have significant implications for the understanding of wettability alteration induced by ions and other chemical agents in the water phase, especially for the understanding of the profound roles of surface charges on the three-phase wettability in a water-decane-silicon dioxide system.

## Abbreviations

CVFF: Consistent valence force field
EOR: Enhanced oil recovery
LAMMPS: Large-scale atomic/molecular massively parallel simulator
MD: Molecular dynamics
OPLS-AA: Optimized potentials for liquid simulations all-atom force field
RDF: Radial distribution function
